# Ebbinghaus, Müller-Lyer, and Ponzo: Three examples of bidirectional space-time interference

**DOI:** 10.3758/s13423-024-02491-7

**Published:** 2024-03-22

**Authors:** Daniel Bratzke

**Affiliations:** Department of Psychology, University of Bremen, Bremen, Germany

**Keywords:** Cross-dimensional interaction, Visual-spatial illusions, Spatial cognition, Time perception

## Abstract

Previous studies have shown interference between illusory size and perceived duration. The present study replicated this space-time interference in three classic visual-spatial illusions, the Ebbinghaus, the Müller-Lyer, and the Ponzo illusion. The results showed bidirectional interference between illusory size and duration for all three illusions. That is, subjectively larger stimuli were judged to be presented longer, and stimuli that were presented longer were judged to be larger. Thus, cross-dimensional interference between illusory size and duration appears to be a robust phenomenon and to generalize across a wide range of visual size illusions. This space-time interference most likely arises at the memory level and supports the theoretical notion of a common representational metric for space and time.

## Introduction

Intuitively, space and time appear to be independent dimensions. In our everyday experience, however, space and time are often correlated. For example, we can travel a longer distance if we have more time. Therefore, the same travel distance could be experienced differently depending on the time spent travelling (see also Cai et al., 2022). Indeed, it has been shown that the processing of space and time information is usually not independent of each other (e.g., Cai & Connell, 2015; Casasanto & Boroditsky, 2008; Eikmeier et al., 2013). In many cases, spatial stimulus attributes affect the perception of time, with larger size leading to longer duration estimates (e.g., Birngruber & Ulrich, 2019; Casasanto & Boroditsky, 2008; Rammsayer & Verner, 2015; Xuan et al., 2007). Initially, space-time interference has been found to be asymmetric (or unidirectional), with space affecting time much more than vice versa (Casasanto & Boroditsky, 2008; Homma & Ashida, 2015). Recently, however, more and more studies have shown bidirectional interference (Bratzke et al., 2024; Cai & Wang, 2022; Cai et al., 2022; Ono & Kawahara, 2007; see also Thomas & Cantor, 1975) or even a reversed asymmetry (Cai & Connell, 2015; Homma & Ashida, 2019).

Two main theoretical models have been used to explain space-time interference: conceptual metaphor theory (CMT; e.g., Lakoff & Johnson, 1980) and “A theory of magnitude” (ATOM; Walsh, 2003). CMT assumes that the mental representation of abstract concepts such as time and number is grounded in mental representations of other concrete dimensions such as space. This means that when thinking or talking about time people use the concrete dimension *space* as a metaphor for the abstract dimension *time* (spatial metaphor account; Boroditsky, 2000). Asymmetric space-time interference has been considered evidence for CMT, as space is used to represent time but time is not used to represent space (Bottini & Casasanto, 2010; Casasanto & Boroditsky, 2008; Janczyk et al., 2023). In contrast to CMT, ATOM assumes that magnitudes like space, number, and time share a common representational metric (Walsh, 2003). Based on this core assumption of ATOM, some researchers have derived the prediction of symmetric space-time interference (e.g., Bottini & Casasanto, 2010). However, Walsh refuted this prediction as being to over-simplistic, not taking into account the developmental history of the dimensions and the tasks used to test cross-dimensional interactions (Walsh, 2013).

Other researchers have focused on the underlying mechanism or processing locus of space-time interference (Bratzke et al., 2024; Cai et al., 2018; Cai et al., 2022; Cai & Connell, 2016; Cai & Wang, 2022; Ono & Kawahara, 2007). Within the framework of internal clock models (e.g., Creelman, 1962; Gibbon et al., 1984; Treisman, 1963; Zakay & Block, 1997), one can ask whether space-time interference arises at the pacemaker stage, which should be reflected in an increase of the space-on-time effect with increasing duration (e.g., Matthews & Meck, 2016; Wearden et al., 1998). Alternatively, space-time interference might arise at the memory stage, when space and time information are maintained simultaneously. Several studies have provided evidence in favor of the latter possibilty, that space-time interference arises from interference at the working memory level and thus at a rather late processing stage (e.g., Cai & Connell, 2016; Cai et al., 2018; Rammsayer & Verner, 2015).

Intriguingly, space-time interference can even occur when spatial differences are implicit (Bottini & Casasanto, 2010; Ma et al., 2012), imagined (Birngruber & Ulrich, 2019), or illusory (Bratzke et al., 2024; Contemori et al., 2023; Ono & Kawahara, 2007). As an example of the latter, Ono and Kawahara (2007) measured the perceived duration of the Ebbinghaus illusion. In this illusion, a central circle is perceived as smaller when surrounded by large compared to small inducer circles. In their Experiment 1, participants had to categorize the duration and size of the central circle after each presentation of the illusion. Ono and Kawahara observed bidirectional space-time interference; that is, subjectively large stimuli were judged to be longer than subjectvely small stimuli, and stimuli that were presented longer were judged to be larger. Since the Ebbinghaus illusion is supposed to be the product of higher-level judgmental processes, the authors concluded that space-time interference arises rather late during visual processing.

Bratzke et al. (2024) replicated Ono and Kawahara’s (2007) finding with a temporal reproduction task and also with another visual size illusion, the horizontal-vertical illusion. Even though the Ebbinghaus and the horizontal-vertical illusion belong to different classes of visual illusions according to some authors (illusion of extent vs. illusion of shape; Coren et al., 1976), they do not cover the whole range of visual-spatial illusions. Two other prominent visual-spatial illusions are the Müller-Lyer and the Ponzo illusions. Axelrod et al. (2017) assessed the Müller-Lyer, Ebbinghaus, Ponzo, and horizontal-vertical illusions together with a non-geometrical (or non-spatial), contrast illusion and observed positive correlations across participants between the magnitudes of the illusions for the spatial illusions but not (or only to a low degree) between the spatial and the contrast illusions. According to the authors, these results suggest that all these illusions share a common mechanism, most probably related to visuospatial integration and scene reconstruction.

Athough Axelrod et al.’s (2017) notion of a common mechanism for visual-spatial illusions seems reasonable, they also observed differences in the relationship between the different illusions. The Ebbinghaus and Müller-Lyer illusions showed the strongest similarities, both in terms of illusion magnitude as well as neural activity, whereas the relationship of the Ebbinghaus illusion with the Ponzo and the horizontal-vertical illusion was weaker. Other research has also pointed to different underlying mechanisms for different spatial illusions (e.g., Cretenoud et al., 2019; Grzeczkowski et al., 2017; Meyer, 1986; Song et al. 2011). Therefore, it seems worthwhile to investigate whether the findings of Ono and Kawahara (2007) and Bratzke et al. (2024) can be replicated and generalized to other visual-spatial illusions. To this end, the present study investigated space-time interference in the Ebbinghaus illusion and two other classic geometrical visual-spatial illusions: the Müller-Lyer and the Ponzo illusions. Ono and Kawahara (2007) already discussed the Müller-Lyer illusion as a candidate for an earlier, low-level locus of space-time interference, referring to previous findings by Lebensfeld and Wapner (1968), who showed that the Kappa effect (i.e., the observation that perceived duration is influenced by apparent distance) can be affected by the Müller-Lyer illusion.

**Fig. 1:**
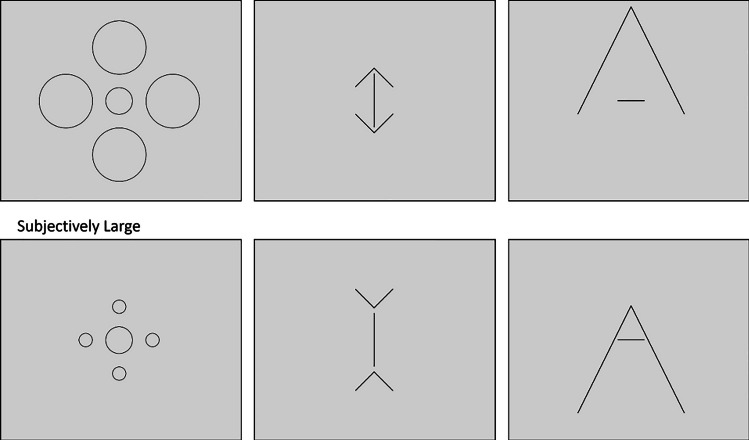
Stimulus configurations for the Ebbinghaus illusion (**Exp. 1, left panels**), the Müller-Lyer illusion (**Exp. 2, middle panels**), and the Ponzo illusion (**Exp. 3, right panels**). For the Ponzo illusion, there were also two configurations with arrows pointing downwards. Note that the “subjectively small” Ebbinghaus configuration consists of larger inducer circles than the “subjectively large” configuration

As Bratzke et al. (2024) used a temporal reproduction paradigm in an online study and this resulted in a rather high outlier rate, and thus, excluded data, the present study used the original categorization task of Ono and Kawahara (2007). Experiment 1 of the present study represents a direct replication of Ono and Kawahara’s Experiment 1. That is, in each trial participants were presented with the Ebbinghaus stimulus and had to categorize the duration as well as the size of the central circle. Experiments 2 and 3 followed the same procedure but used the Müller-Lyer and Ponzo illusions, respectively (see Fig. 1).

## Method

### Participants

A power analysis for an effect size Cohen’s *f* = .056 (based on the smallest of the two main effects of illusory size on duration judgments of Ono and Kawahara, 2007 in their Experiment 2), with power = .90 and alpha = .05 yielded a required sample size of *N* = 36. The final sample sizes were 63 participants in Experiment 1 (mean age: 27.5 years, *SD* = 11.6, 45 female, 16 male, one non-binary, one with no report), 46 participants in Experiment 2 (mean age: 28.7 years, *SD* = 12.0, 32 female and 14 male), and 49 participants in Experiment 3 (mean age: 26.1 years, *SD* = 10.9, 29 females, 18 males, and two non-binary). All participants had normal or corrected-to-normal visual acuity and provided informed consent before data collection.

### Apparatus and stimuli

All experiments were online experiments and run on the participants’ individual computers. They were created in PsychoPy (Peirce et al., 2019) and hosted by Pavlovia (https://pavlovia.org). Visual angles of the stimuli varied across participants due to individual screen sizes. All stimuli were presented in black on a gray background, and all target stimuli were presented in the center of the screen. For all illusions, the sizes or lengths of comparison stimuli for the spatial judgments were evenly distributed around the target size or length, respectively. They were labeled from “1” for the smallest or shortest to “8” for the largest size or longest length. The following visual angles refer to a 15.6-in. screen and a viewing distance of 50 cm. The Ebbinghaus stimuli consisted of four outer inducer circles and one central target circle. The diameter of the inducer circles was 4.0° in the “subjective small” condition and 1.0° in the “subjective large” condition. The central circle always had a diameter of 2.0°. The distance between the inducer circles and the central circle was 1.0°. There were eight comparison circles, which ranged from 1.65° to 2.35°. Comparison circles were presented in ascending order from left to right in two rows of four circles each. The Müller-Lyer stimuli consisted of two outer right angle inducer arrows (with an invisible base of 2.5°) pointing either upwards or downwards and one central vertical target line (4.0°). The distance between the arrowhead and the vertical line was about 0.4°. Comparison stimuli for the Müller-Lyer illusion consisted of eight lines between 3.65° and 4.35° and were presented from left to right in ascending order. The Ponzo stimuli consisted of an outer inducer arrow (an isosceles triangle with an invisible base of 8.0° and a height of 8.0°) pointing upwards or downwards and one central horizontal target line (2.0°). The distance between the arrowhead and the target line was either 2.6° (“subjectively large” inducer) or 7.0° (“subjectively small” inducer). Comparison stimuli for the Ponzo illusion were eight horizontal lines between 1.65° and 2.35°, which were arranged in ascending order from left to right in two rows of four lines each.

### Task and procedure

The task and procedure were the same as in Experiment 1 of Ono and Kawahara (2007). Each experiment started with a practice block, in which participants learned to categorize the duration of target stimuli. No inducer stimuli were presented in this phase. Each practice trial started with the presentation of a central fixation cross for 2,000 ms. Then the target stimulus was presented for one of four possible durations (100, 200, 300, and 400 ms). Participants were asked to categorize the target duration into four possible categories, ranging from “1” for short to “4” for long, by pressing the corresponding key on the computer keyboard. Each participant completed 60 practice trials in total. In the subsequent test block, each trial started with a presentation of the inducers for 1,500 ms. Then, the central target circle appeared for 150 or 350 ms, together with the inducers. The inducers remained on the screen for another 1,000 ms thereafter. Then, participants had to categorize the duration of the central target stimulus by pressing one of the four possible keys (1–4). With the participant’s response, the eight comparison stimuli appeared on the screen until the participant provided their size or length estimate by pressing one of eight keys (1–8). The experimental block consisted of 120 trials in total.

## Results

**Fig. 2:**
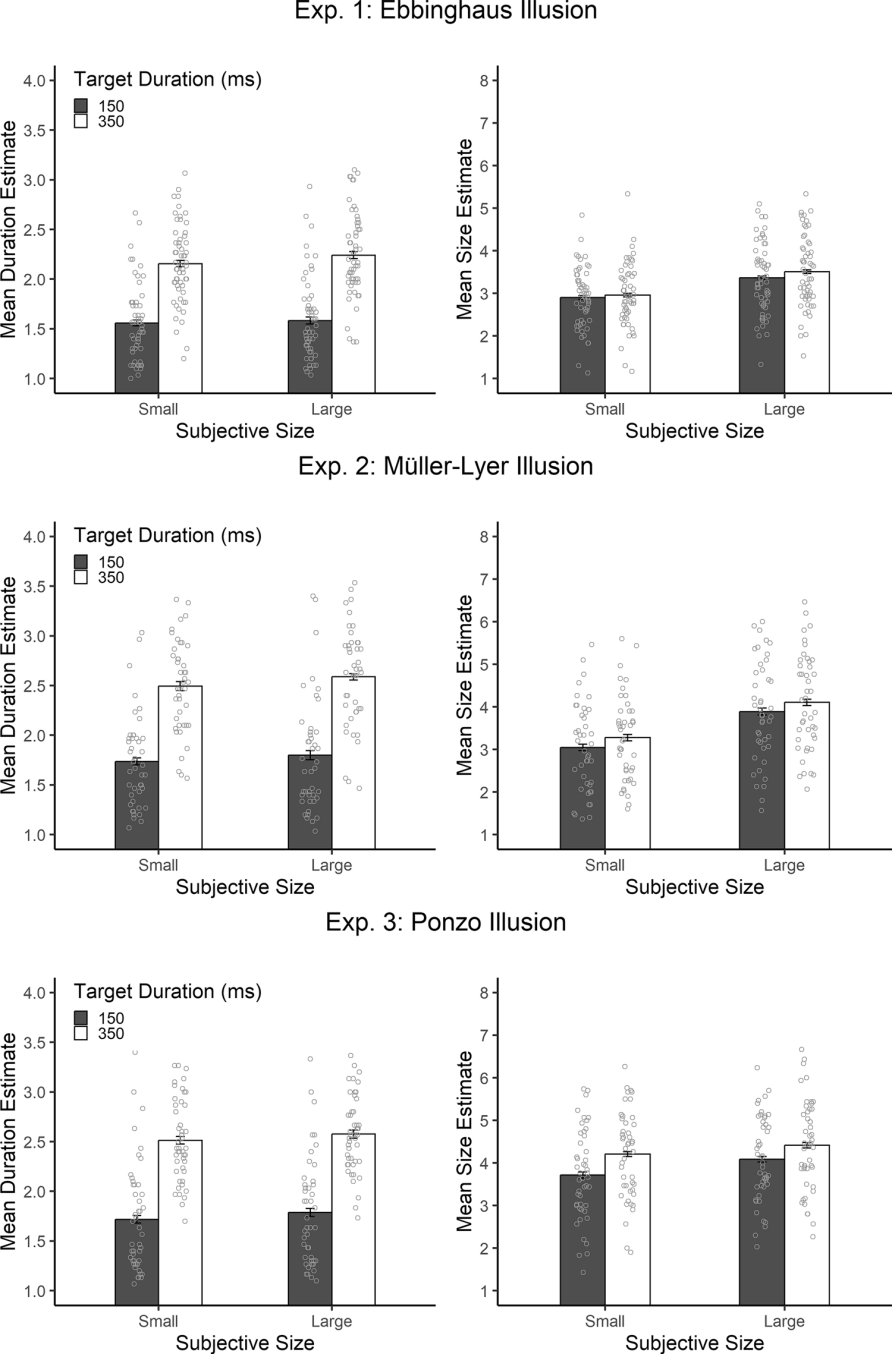
Mean duration estimates (**left**) and mean size estimates (**right**) as a function of subjective size and target duration in the Ebbinghaus illusion (**Exp. 1, top**), the Müller-Lyer illusion (**Exp. 2, middle**), and the Ponzo illusion (**Exp. 3, bottom**). Gray circles represent individual mean estimates, and error bars represent ± 1 within-subjects *SE* according to Morey (2008)

Data of participants whose mean duration and/or size estimate deviated more than ± 2.5 *SD*s from the overall mean in the respective experiment were excluded from analyses (six participants in Experiment 1, one participant in Experiment 2, and two participants in Experiment 3). One additional participant in Experiment 2 was excluded because of showing no variability in duration and size estimates. The results of Experiments 1–3 are depicted in Fig. 2. Following Ono and Kawahara (2007), separate ANOVAs with the within-subject factors target duration (150 vs. 350 ms), subjective size (subjectively small vs. subjectively large), and the between-subjects factor illusion type (Ebbinghaus, Müller-Lyer, and Ponzo) were conducted for mean duration and size estimates. For post hoc pairwise comparisons, *p*-values were Holm-Bonferroni adjusted. If participants were able to veridically estimate the target duration, their mean duration estimates should be 1.5 for the 150 ms and 3.5 for the 350 ms target duration condition. A veridical estimation of the target stimulus would be reflected in a mean size estimate of 4.5.

### Duration estimates

The overall mean duration estimate was 2.05, which reflects an understimation of the average target duration (250 ms, *p* < .001). The ANOVA revealed a significant main effect of target duration, *F*(1, 146) = 464.90, *p* < .001, η_p_^2^ = .76. The mean duration estimates were 2.41 (significantly different from 3.5, *p* < .001) for the long target duration, and 1.69 (significantly different from 1.5, *p* < .001) for the short target duration. More importantly, there was also a significant main effect of subjective size on duration estimates, *F*(1, 146) = 24.25, *p* < .001, η_p_^2^ = .15. Mean duration estimates were longer in the subjectively large (2.08) than in the subjectively small (2.01) condition. The main effect of illusion type was also significant, *F*(2, 146) = 7.97, *p* =.001, η_p_^2^ = .10. Duration estimates were on average longer for the Müller-Lyer and the Ponzo illusions (both 2.15) than for the Ebbinghaus illusion (1.88). There was no significant interaction between target duration and subjective size, *F*(2, 146) = 2.62, *p* =.108, η_p_^2^ = .02. The between-subjects factor illusion type did not significantly interact with any of the within-subject factors, both *p*s ≥ .082, and there was also no three-way interaction, *F*(2, 146) = 1.73, *p* =.258, η_p_^2^ = .02.

### Size estimates

Individual Spearman correlations between size and duration estimates ranged from -.17 to 1, with a mean of .20 (significantly different from zero, *p* < .001). The ANOVA indicated a main effect of illusion type, *F*(2, 146) = 14.08, *p* < .001, η_p_^2^ = .16, with larger size estimates for the Ponzo illusion (4.10) than for the Müller-Lyer (3.60) and the Ebbinghaus illusions (3.28). Post hoc pairwise *t*-tests indicated that size estimates differed between all illusions (all *p*s < .001) and that participants underestimated the objective target size in all illusions (all *p*s < .001). As expected, mean size estimates were affected by subjective size, *F*(1, 146) = 103.09, *p* < .001, η_p_^2^ = .41. The illusion was largest for the Müller-Lyer (0.83), followed by the Ebbinghaus (0.51) and the Ponzo illusions (0.29), *F*(2, 146) = 8.06, *p* < .001, η_p_^2^ = .10. Post hoc pairwise *t*-tests indicated that the Müller-Lyer illusion differed from the Ebbinghaus (*p* = . 048) and the Ponzo (*p* = .002) illusions, wheras the Ebbinghaus and the Ponzo illusions did not differ from each other (*p* = .055). Most crucially, mean size estimates were also affected by target duration, *F*(1, 146) = 128.41, *p* < .001, η_p_^2^ = .47. Size estimates were larger for the long (3.75) than for the short (3.50) target duration. This effect was modulated by illusion type, *F*(2, 146) = 18.44, *p* < .001, η_p_^2^ = .20. The effect of target duration (long minus short) was largest for the Ponzo illusion (0.41), followed by the Müller-Lyer illusion (0.22), and smallest for the Ebbinghaus illusion (0.10). Post hoc pairwise *t*-tests indicated that the effect of target duration differed between all illusions (all *p*s ≤ .008) The interaction between target duration and subjective size was not significant, *F*(2, 146) = 0.62, *p* = .434, η_p_^2^ < .01. The three-way interaction, however, was significant, *F*(2, 146) = 4.07, *p* = .019, η_p_^2^ = .05. Descriptively, target duration and subjective size interacted differently (duration effect for subjectively large stimuli minus duration effect for subjectively small stimuli) between the different illusions, with slightly overadditive effects for the Ebbinghaus illusion (0.09), rather additive effects for the Müller-Lyer illusion (-0.01), and slightly underadditive effects for the Ponzo illusion (-0.16). Separate ANOVAs for the different illusion types, however, did not show any significant target duration by subjective size interaction (Ebbinghaus: *p* = .083, Müller-Lyer: *p* = .820, Ponzo: *p* = .052).

## Discussion

The present study investigated space-time interference in three visual size illusions, the Ebbinghaus, the Müller-Lyer, and the Ponzo illusions. The results replicated previous observations of bidirectional space-time interference by Ono and Kawahara (2007) and Bratzke et al. (2024). That is, a larger subjective size led to longer duration estimates, and a longer duration led to larger size estimates. This pattern appeared for all illusions, even though there were some differences in the strength of the illusion between the three illusion types (Müller-Lyer > Ebbinghaus = Ponzo). Taken together with the two previous studies, bidirectional space-time interference for illusory size seems to be reliable and to generalize across a wide range of visual-spatial illusions.

With regard to the locus of space-time interference, the interaction pattern between size and target duration can provide useful information. For example, an effect of size on the rate of an internal clock’s pacemaker should increase with target duration (e.g., Matthews & Meck, 2016; Wearden et al., 1998). The present study showed no such interaction between subjective size and target duration for duration estimates. This is consistent with the results of Experiment 1 of Ono and Kawahara (2007; but see their Experiment 2 for an overadditive interaction). Even though the range of target durations used in the present study, as well as Ono and Kawahara’ study, is rather limited (150–350 ms), there is no evidence that illusory size could affect the rate of an internal clock’s pacemaker.

Visual size illusions are often considered to arise rather late during visual processing from high-level feedback-mediated processing, such as spatial context integration and scene construction (Axelrod et al., 2017; Schmidt et al., 2016; Song et al., 2011). In line with this assumption, visual size illusions have been shown to take some time to become fully effective (Schmidt et al., 2016; Schmidt & Haberkamp, 2016; Schulz, 1991), and to diminish with longer viewing times (> 0.3–1 s; Bressan & Kramer, 2021; de Brouwer et al., 2014). Previous research also suggests differences between visual-size illusions in their temporal dynamic. In particular, the Ebbinghaus illusion appears to be unique in that it is reversed in its very early processing (Schmidt et al., 2016). However, the typical Ebbinghaus illusion can be observed, even when the Ebbinghaus stimulus is presented for a very short time (12 s; Schmidt et al., 2016), suggesting that processing of the illusion stimulus continues after its offset. Based on these previous results, one would probably not expect the illusions to differ substantially between the two target durations (150–350 ms). However, the significant three-way interaction for size estimates suggests that the Ponzo illusion developed slightly faster (or dimished earlier) and the Ebbinghaus illusion developed slightly slower than the Müller-Lyer illusion. As for the locus of space-time interference, it is conceivable that the processing of time information interferes with the feedback processes that are critical for the size illusions. However, it is not readily apparent how such an interference would occur at this stage of processing, nor why it would be bidirectional.

The most likely explanation for space-time interference in visual size illusions appears to be memory interference when size and duration information are concurrently held in working memory (e.g., Cai & Connell, 2015, 2016; Cai et al., 2018), likely facilitated by a common mental metric for space and time information (Walsh, 2003). This memory account is essentially supported by two previous observations. First, temporal reproduction studies have shown that the two dimensions affect each other when the size information is presented during encoding, but do not affect each other when it is presented during reproduction (Cai & Connell, 2016; Rammsayer &Verner, 2015). Second, Cai et al. (2018) observed that size affected temporal reproductions only when the relevant size information was cued before the retrieval of the duration information.

Furthermore, the present bidirectional space-time interference argues against the spatial metaphor account derived from CMT (e.g., Boroditsky, 2000) as an explanation for interference between size and duration. The present evidence, however, does not rule out the spatial metaphor account in general, as it may be suitable for dimensions that rely more heavily on linguistic processing (e.g., number processing; see, e.g., Walsh, 2013) as well as other types of space-time interference (e.g., between lateral space and deictic time; Janczyk et al., 2023). Some authors have suggested that the two main theoretical accounts for explaining space-time interactions, ATOM and CMT, can complement each other in explaining cross-dimensional interference at different processing levels (Winter et al., 2015; see also Walsh, 2013).

In all experiments of the present study, participants underestimated the duration as well as the size of the target stimulus, even though the short duration was slightly overestimated (consistent with the typical central tendency effect in timing performance, e.g., Bausenhart et al., 2016; Jazayeri & Shadlen, 2010; Vierordt, 1868; Zimmermann, & Cicchini, 2020). Similar underestimations can also be found in all three experiments of Ono and Kawahara’s (2007) study (as estimated from their Tables 2–4). In fact, the underestimation of target size is also in line with the previous result that the mere existence of inducers can reduce apparent target size in the Ebbinghaus illusion (Roberts et al., 2005). Nevertheless, memory-related subjective shortening might have also played a role in the present praradigm because duration and size estimates were not collected until at least 1 s after each illusion stimulus (but see Wearden et al., 2002, for evidence that judgments of physical length are less prone to subjective shortening than duration judgments).

In the present study, the different types of illusions were studied between subjects. This choice limits the comparison of effect sizes between the different illusions and makes it impossible to examine the correlation between space-time interference across the different illusions. While such an examination could provide further insights into the underlying mechanism of space-time interference in these different illusions (see also Cretenoud et al., 2019), a between-subject design was chosen to minimise the time and attentional demands on participants and thus ensure their compliance in the present online experimentation context.

In sum, the present study replicated and extended previous observations of space-time interference in visual size illusions. Bidirectional cross-dimensional interference was shown for the Ebbinghaus, the Müller-Lyer, and the Ponzo Illusion. Thus, it seems fair to conclude that space-time interference is a robust phenomenon even when size differences are illusory and that this interference generalizes widely across different visual size illusions.

## Declarations

### Open practices statement

The datasets generated and/or analyzed during the current study are available from the corresponding author on reasonable request. This study was not preregistered.
